# Nicotinamide adenine dinucleotide as a photocatalyst

**DOI:** 10.1126/sciadv.aax0501

**Published:** 2019-07-19

**Authors:** Jinhyun Kim, Sahng Ha Lee, Florian Tieves, Caroline E. Paul, Frank Hollmann, Chan Beum Park

**Affiliations:** 1 Department of Materials Science and Engineering, Korea Advanced Institute of Science and Technology (KAIST), 335 Science Road, Daejeon 305-701, Republic of Korea.; 2 Department of Biotechnology, Delft University of Technology, Van der Maasweg 9, 2629 HZ Delft, Netherlands.

## Abstract

Nicotinamide adenine dinucleotide (NAD^+^) is a key redox compound in all living cells responsible for energy transduction, genomic integrity, life-span extension, and neuromodulation. Here, we report a new function of NAD^+^ as a molecular photocatalyst in addition to the biological roles. Our spectroscopic and electrochemical analyses reveal light absorption and electronic properties of two π-conjugated systems of NAD^+^. Furthermore, NAD^+^ exhibits a robust photostability under UV-Vis-NIR irradiation. We demonstrate photocatalytic redox reactions driven by NAD^+^, such as O_2_ reduction, H_2_O oxidation, and the formation of metallic nanoparticles. Beyond the traditional role of NAD^+^ as a cofactor in redox biocatalysis, NAD^+^ executes direct photoactivation of oxidoreductases through the reduction of enzyme prosthetic groups. Consequently, the synergetic integration of biocatalysis and photocatalysis using NAD^+^ enables solar-to-chemical conversion with the highest-ever-recorded turnover frequency and total turnover number of 1263.4 hour^−1^ and 1692.3, respectively, for light-driven biocatalytic trans-hydrogenation.

## Teaser

Nicotinamide adenine dinucleotide functions as a molecular photocatalyst, distinct from its pivotal roles in biological processes.

## INTRODUCTION

Nicotinamide adenine dinucleotide (NAD^+^) is a vital cofactor that functions as an electron carrier in cellular energy transduction (*1*). The cytosolic and mitochondrial pools of NAD^+^ modulate the activity of compartment-specific metabolic pathways, such as glycolysis in the cytoplasm and tricarboxylic acid cycle in the mitochondria. In the cytoplasm, NAD^+^ is reduced to NADH through glycolysis by glyceraldehyde-3-phosphate dehydrogenase. The cytosolic NADH is transported into mitochondria and then oxidized by complex I (NADH:ubiquinone oxidoreductase) for transferring electrons to the electron transport chain. This electron relay drives the chemiosmotic synthesis of adenosine triphosphate as an energy storage molecule. Beyond the role for redox shuttling, NAD^+^ is a cosubstrate for a variety of redox enzymes, such as poly[adenosine diphosphate (ADP)–ribose] polymerases (PARPs) and sirtuins (SIRTs) (*2*, *3*). Some members of the PARP and SIRT families cleave NAD^+^ to use the ADP-ribose moiety in the interest of genomic integrity, mitochondrial biogenesis, improved metabolic efficiency, and life-span extension. In addition to intracellular roles, NAD^+^ functions as a neurotransmitter and neuromodulator in the peripheral nervous system after it is secreted from neurons in blood vessels, the urinary bladder, and the colon (*4*). For instance, extracellular NAD^+^ tunes the release of other neurotransmitters (e.g., norepinephrine) in blood vessels, inhibits spontaneous smooth muscle contractions in urinary bladders, and causes membrane hyperpolarization and relaxation in colons.

**Fig. 1:**
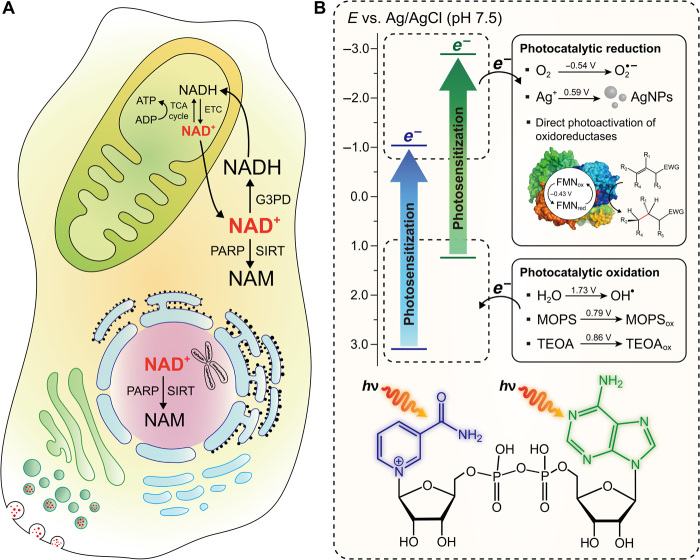
Illustrative comparison of biological and photocatalytic functions of NAD^+^. (**A**) Cellular NAD^+^ metabolism in different cellular compartments for adenosine triphosphate (ATP) synthesis, genomic integrity, mitochondrial biogenesis, improved metabolic efficiency, and life-span extension. G3PD, glyceraldehyde-3-phosphate dehydrogenase; TCA, tricarboxylic acid; ETC, electron transport chain; NAM, nicotinamide. (**B**) Energy diagram for NAD^+^-sensitized photocatalytic redox reactions [e.g., reduction of O_2_ to O_2_^•−^, formation of AgNPs, direct photoactivation of *Ts*OYE (OYE homolog from *Thermus scotoductus*), oxidation of H_2_O to OH^•^, oxidation of MOPS, and oxidation of triethanolamine (TEOA)]. FMN, flavin mononucleotide; EWG, electron-withdrawing group.

Here, we report a newly found function of NAD^+^ as a molecular photocatalyst, distinct from the biological roles of NAD^+^ as a cofactor, cosubstrate, neurotransmitter, and neuromodulator (Fig. 1). A photocatalyst absorbs light, excites its electrons (or holes) to higher electronic levels, and makes the excited charges participate in a photoredox reaction. Solar energy has emerged as a clean and inexhaustible resource; thus, molecular photocatalysts have been widely applied for solar-driven redox chemistry [e.g., organic synthesis (*5*), hydrogen production (*6*), and CO_2_ reduction (*6*)]. Furthermore, photocatalysis has been recently combined with biocatalysis to expand the scope of cascade-type syntheses (*7*). NAD^+^ contains π-conjugated systems (i.e., nicotinamide and adenine), at which electrons are delocalized in circular π bonds from the overlap of hybridized atomic *p*_z_ orbitals. Upon photosensitization of NAD^+^, these electrons can be excited to energetically higher levels, obtaining a reducing power enough to reduce adjacent molecules or ions. We have conducted proof-of-concept experiments to demonstrate that NAD^+^ can perform photocatalytic redox reactions, such as H_2_O oxidation coupled with O_2_ reduction and the growth of silver nanoparticles (AgNPs) from Ag^+^ ion reduction. Furthermore, beyond the function of NAD^+^ as a cofactor, photoactivated NAD^+^ can directly activate redox enzymes such as ene-reductases [from the Old Yellow Enzyme (OYE) family] through the electron transfer from NAD^+^ to the prosthetic flavin moiety. Subsequently, the redox enzyme catalyzes the stereoselective hydrogenation of activated C═C bonds.

## RESULTS

### Electronic property and photostability of NAD^+^

To comprehend the origin of NAD^+^’s photocatalytic activity, we investigated its electronic properties using ultraviolet-visible (UV-Vis) spectroscopy. NAD^+^ exhibited a characteristic absorption peak at around 260 nm that stems from the π-π* electronic transition (*8*) of nicotinamide and adenine (fig. S1A). Upon shining light (λ, ~260 nm) on NAD^+^, π electrons in the nicotinamide and adenine moieties were excited to the lowest unoccupied molecular orbitals (LUMO) or higher energy levels, making NAD^+^ a potent reductant. Next, we obtained cyclic voltammograms of NAD^+^ and ferrocene to estimate the energy levels of LUMO and the highest occupied molecular orbital (HOMO) of NAD^+^. As displayed in fig. S1 (B to D), NAD^+^ exhibited an onset of reduction wave at around −0.94 V (versus Ag/AgCl) and that of oxidation wave at around 1.20 V (versus Ag/AgCl); the cathodic and anodic currents originated from the reduction of nicotinamide (*9*) and the oxidation of adenine (*10*), respectively. On the basis of the formal potential of ferrocene (fig. S1E) and the onset potentials of NAD^+^, we found that the energies of LUMO_Nicotinamide_ and HOMO_Adenine_ are −0.90 and 1.24 V (versus Ag/AgCl), respectively. In addition, HOMO_Nicotinamide_ of 3.23 V (versus Ag/AgCl) and LUMO_Adenine_ of −2.89 V (versus Ag/AgCl) were estimated on the basis of the HOMO-LUMO gap of 4.13 eV (300 nm) from the absorption onset wavelength (fig. S1F). We verified a negligible photodegradation of NAD^+^ under illumination (λ, 260 to 900 nm; *P*_260–900 nm_, 200 mW cm^−2^; *P*_260–300 nm_, 10 mW cm^−2^), as shown in fig. S2. This excellent photostability is a highly desirable property of molecular photocatalysts because of photobleaching and instability issues of many molecular photocatalysts (*7*, *11*).

### NAD^+^-driven photocatalytic H_2_O oxidation and O_2_ reduction

**Fig. 2:**
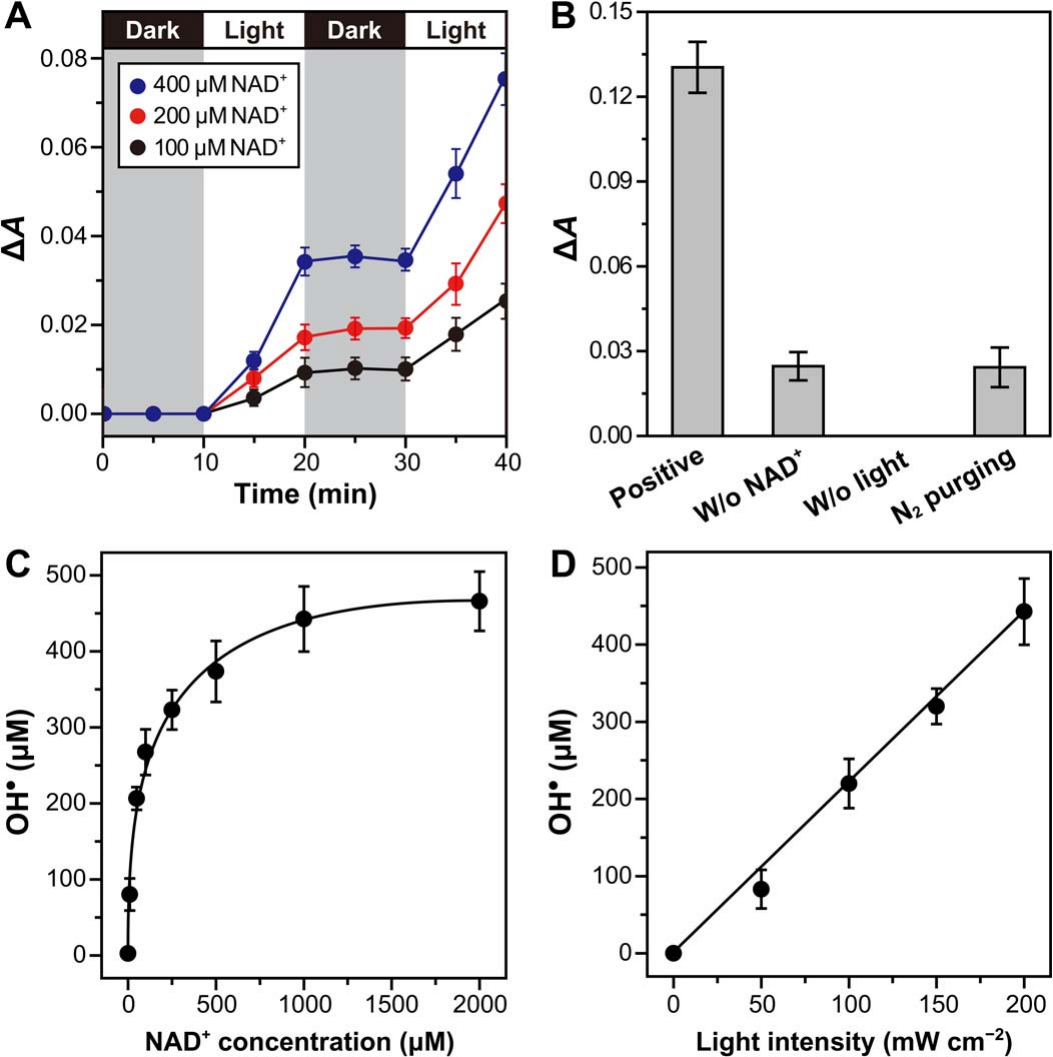
NAD^+^-sensitized reduction of O_2_and oxidation of H_2_O. (**A**) Absorbance changes of NBT solution at 560 nm with varying concentration of NAD^+^. Δ*A*(*t*) ≡ *A*(*t*) − *A*(*t* = 0 min). The background signal [displayed in (B)] was not subtracted from Δ*A*(*t*). Reaction condition: NAD^+^ and NBT in an O_2_-purged sodium phosphate buffer (50 mM, pH 7.5) under irradiation (xenon lamp: λ, 260 to 900 nm and *P*, 200 mW cm^−2^). (**B**) A series of control experiments for each reaction component (i.e., 400 μM NAD^+^, light, and O_2_) in the photochemical formation of O_2_^•−^. Δ*A* ≡ *A*(30 min) − *A*(0 min). Reaction conditions: NAD^+^ and NBT in an O_2_-purged sodium phosphate buffer (50 mM, pH 7.5) under irradiation (xenon lamp: λ, 260 to 900 nm and *P*, 200 mW cm^−2^). (**C**) Effect of NAD^+^ concentration on OH^•^ formation for 30-min irradiation (200 mW cm^−2^). (**D**) Dependency of OH^•^ formation on the light intensity (*t* = 30 min). Reaction conditions: 1 mM NAD^+^ and Tris in an O_2_-purged sodium phosphate buffer (50 mM, pH 7.5). All reported values represent means ± SD (*n* = 3).

Building on the electronic properties and photostability of NAD^+^, we investigated its capability to photocatalytically reduce O_2_ to superoxide ion (O_2_^•−^) because its reduction potential (O_2_/O_2_^•−^, *E*_red_ = −0.54 V versus Ag/AgCl) (*12*) is more positive than LUMO_Nicotinamide_ of −0.90 V (versus Ag/AgCl) and LUMO_Adenine_ of −2.89 V (versus Ag/AgCl). We analyzed the formation of superoxide ion (O_2_^•−^) using a nitro blue tetrazolium (NBT) assay; the reduction of NBT by O_2_^•−^ forms NBT formazan, which can be monitored spectrophotometrically at 560 nm (*13*). As shown in Fig. 2A and fig. S3A, O_2_^•−^ formation was achieved only under irradiation with light (λ, 260 to 900 nm), and the ion’s concentration increased with the increasing concentration of NAD^+^ and light intensity (*P*, 0 to 200 mW cm^−2^). The photochemical formation of O_2_^•−^ was also triggered under filtered illumination (λ, 260 to 390 nm; *P*, 0 to 20 mW cm^−2^) but not under visible–near-infrared (Vis-NIR) illumination (λ, 360 to 900 nm; *P*, 0 to 200 mW cm^−2^). It is ascribed to the negligible photoactivation of NAD^+^ because NAD^+^ does not absorb light longer than 300 nm. Control experiments in the absence of NAD^+^ or O_2_ resulted in a background signal (Fig. 2B); NBT, phosphate ions, and UV light were necessities for reduction of NBT to NBT formazan (fig. S3B). According to the literature (*14*), the exposure of NBT to UV light increases the redox potential of NBT, transforming it into a stronger oxidant to extract electrons directly from neighboring molecules other than from O_2_^•−^.

In the photochemical reduction of O_2_, an anodic reaction should occur as a counterpart to accomplish a redox reaction. Because we did not use an artificial electron donor for the NAD^+^-sensitized formation of O_2_^•−^, we hypothesized that photoactivated NAD^+^ can oxidize H_2_O. The products of H_2_O oxidation with corresponding oxidation potentials at pH 7.5 are as follows (Eqs. 1 to 3) (*15*)

$$H_{2}O\to {\text{OH}}^{•}+(H^{+}+e^{-}),E_{\text{ox}}=1.73V(\text{versus}\;\text{Ag}/\text{AgCl})$$ (1)

$$2H_{2}O\to H_{2}O_{2}+2(H^{+}+e^{-}),E_{\text{ox}}=1.11V(\text{versus}\;\text{Ag}/\text{AgCl})$$ (2)

$$2H_{2}O\to O_{2}+4(H^{+}+e^{-}),E_{\text{ox}}=0.58V(\text{versus}\;\text{Ag}/\text{AgCl})$$ (3)

We measured hydroxyl radicals (OH^•^) because the one-electron–one-proton oxidation of water (Eq. 1) is kinetically more favorable than two-electron–two-proton or four-electron–four-proton oxidation of water (Eqs. 2 and 3). To determine the concentration of OH^•^, we performed a colorimetric assay using tris(hydroxymethyl)aminomethane (Tris) and Nash’s reagent (*12*, *16*, *17*); Tris reacts with OH^•^ to yield formaldehyde (stoichiometric ratio of 1:1:1), and the formaldehyde can be quantitatively monitored using Nash’s reagent (fig. S4). We confirmed that both NAD^+^ and light were requisites for photochemical formation of OH^•^ (fig. S5A); NAD^+^ concentration and light intensity increased the concentration of the radical (Fig. 2, C and D). However, the filtered light (λ, 360 to 900 nm; *P*, 200 mW cm^−2^) did not prompt the NAD^+^-driven generation of OH^•^ due to the negligible photoexcitation of NAD^+^ (fig. S5A). Because OH^•^ can be formed in the course of O_2_ reduction (fig. S5B) (*18*), we further conducted an additional experiment of photochemical OH^•^ generation under N_2_-rich conditions. The radical’s amount was ca. 68% of that generated under O_2_-rich conditions (fig. S5C), indicating that the contribution of H_2_O oxidation is greater than that of O_2_ reduction. On the basis of the widely accepted mechanism of molecular photoredox catalysis (*5*), a possible mechanism for O_2_ reduction and H_2_O oxidation is suggested to be a combination of oxidative and reductive quenching cycles (fig. S5, D and E). Light absorption of NAD^+^ transforms it into a photoactivated state, [NAD^+^]^*^. In an oxidative quenching process, O_2_ is reduced to O_2_^•−^ by receiving electrons from [NAD^+^]^*^. Subsequently, the oxidized catalyst, [NAD^+^]^•+^, oxidizes H_2_O to OH^•^ and returns to the original state of NAD^+^. In a reductive quenching stage, the reduced catalyst, [NAD^+^]^•−^, is formed through H_2_O oxidation, which is then transformed into its original state via O_2_ reduction. The HOMO and LUMO levels of NAD^+^ makes [NAD^+^]* thermodynamically favorable for photocatalytic production of O_2_^•−^ and OH^•^.

### Metal ion reduction by photoexcited NAD^+^

We investigated the possibility of photochemically reducing Ag^+^ ions to AgNPs by NAD^+^ to procure additional evidence of NAD^+^-driven photocatalytic redox reactions. According to the literature (*19*), a photocatalyst transfers its photoexcited electrons to Ag^+^ ions, which become Ag atoms and form seed nuclei. These nuclei function as templates for the growth of AgNPs based on the Lifshitz-Slyozov-Wagner theory or the autocatalytic reduction-nucleation process proposed by Finke and Watzky. Because the interaction between electron donor (i.e., NAD^+^) and acceptor (i.e., Ag^+^ ion) plays an important role in redox catalysis, we used UV-Vis spectroscopy to examine the cation-π interaction between Ag^+^ ions and the π-conjugated moieties of NAD^+^. The absorbance of NAD^+^ at ca. 260 nm gradually decreased with the concentration of Ag^+^ ions (fig. S6A), which indicates the alteration of the π-π* transition of the nicotinamide and adenine moieties of NAD^+^ through noncovalent ion-quadrupole interaction. The increase in concentration of Ag^+^ ions (from 0 to 10 μM) caused the spectrophotometric change in the absorbance at around 210 nm (fig. S6B).

After identifying the favorable noncovalent interaction between NAD^+^ and Ag^+^ ions, we exposed a solution of NAD^+^ and silver nitrate (AgNO_3_) in deionized water to light from a solar simulator (λ, 260 to 900 nm; *P*_260–300 nm_, 5 mW cm^−2^; *P*_260–900 nm_, 100 mW cm^−2^). We did not use additional sacrificial electron donors because H_2_O is an electron donor of photoactivated NAD^+^. As displayed in fig. S6 (C and D), we observed a localized surface plasmon resonance (LSPR) band of the AgNPs, which shows the resonant harmonic oscillation of surface electrons in AgNPs upon incident electromagnetic radiation on AgNPs (*20*). In contrast, a negligible LSPR band was detected in the absence of NAD^+^ or light (fig. S6, E to H). The high-resolution transmission electron microscopic image of thus-synthesized AgNPs in fig. S6I shows that the AgNPs were quasi-spherical with a diameter of 15.8 ± 3.8 nm.

**Fig. 3:**
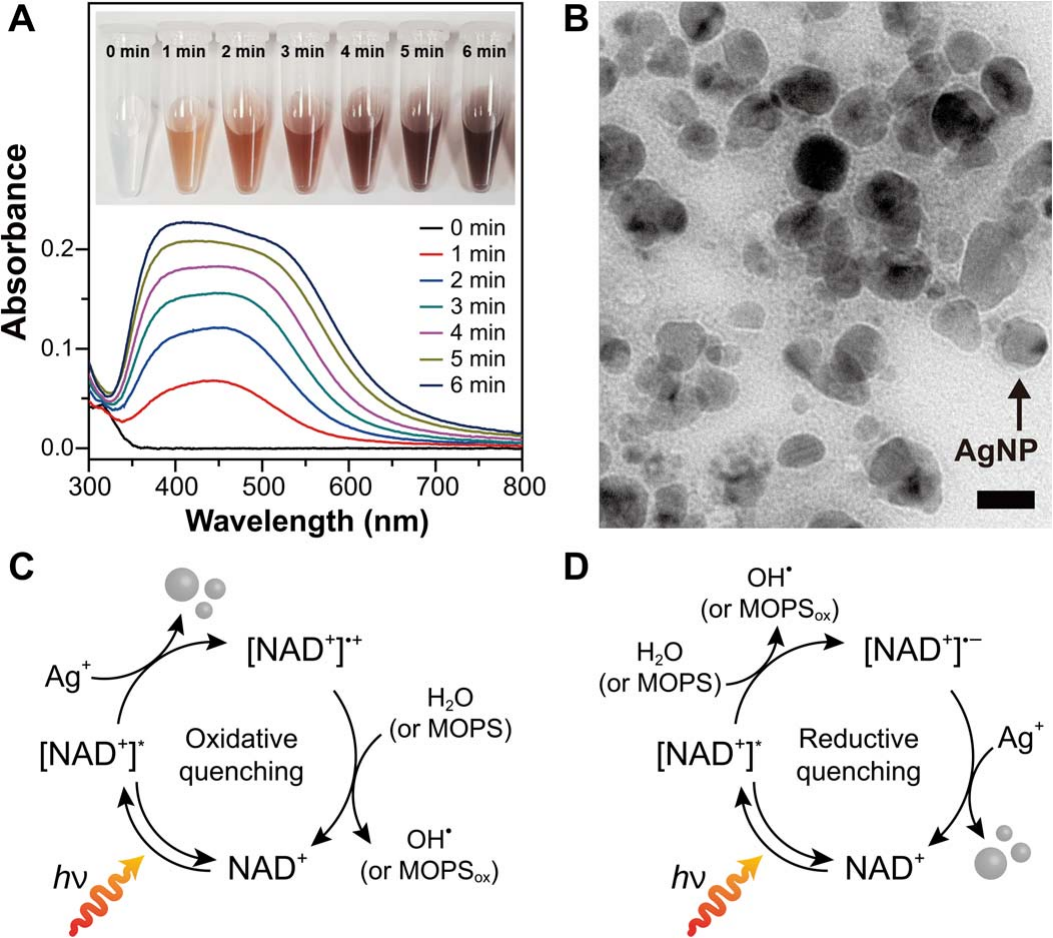
NAD^+^-driven photocatalytic formation of AgNPs. (**A**) Spectrophotometric changes in the absorbance of AgNP solution. UV-Vis absorption spectra were obtained after 10-fold dilution of samples. Inset: A photograph showing the color change of AgNP solutions containing NAD^+^. Photo credit: Jinhyun Kim, Korea Advanced Institute of Science and Technology. Reaction conditions: 0.5 mM NAD^+^ and 1 mM AgNO_3_ in a N_2_-purged MOPS buffer (50 mM, pH 7.5) under illumination (xenon lamp: *P*_260–900 nm_, 100 mW cm^−2^) at 293.15 K. (**B**) High-resolution transmission electron microscopy image of AgNPs synthesized using NAD^+^ for 1-min illumination. Scale bar, 20 nm. (**C** and **D**) Plausible photocatalytic cycles for the production of AgNPs driven by NAD^+^ through (C) an oxidative quenching process or (D) a reductive quenching process. Regardless of the processes, Ag^+^ ions are reduced by receiving photoexcited electrons from NAD^+^, whereas H_2_O (or MOPS) donates its electrons to NAD^+^.

After observing the rather slow formation of AgNPs, we hypothesized that the use of a sacrificial electron-supplying agent may improve the formation rate of AgNPs if the oxidation kinetics of an electron donor is faster than those of water by photoactivated NAD^+^. Note that a kinetic bottleneck of water oxidation could be a cause of the low reduction rate of the counterpart, which is a well-known issue in photo(electro)catalysis (*21*, *22*). We used 3-(*N*-morpholino)propanesulfonic acid (MOPS) as a model electron-supplying agent; it has a tertiary amine that can provide its electrons to an excited photocatalyst. We observed its oxidation potential at 0.79 V versus Ag/AgCl (fig. S7A), which indicates that the electron transfer from MOPS to photoactivated NAD^+^ is thermodynamically favorable. As displayed in Fig. 3A, the intensity of the LSPR band in a MOPS buffer increased more than 10 times faster than that in H_2_O (fig. S6C; the absorption spectra of AgNPs in a MOPS buffer were obtained after 10-fold dilution of samples). The concentration of the AgNPs synthesized in a MOPS buffer increased with the irradiation time (fig. S7B; see the detailed analytical procedures in the Materials and Methods section). In addition, both NAD^+^ and light were required to obtain a distinctive LSPR band of AgNPs (fig. S7, C and D). The diameter of AgNPs synthesized in a MOPS buffer (17.0 ± 4.8 nm; Fig. 3B and fig. S7E) with 1-min irradiation was comparable to that of the plasmonic nanoparticles prepared in H_2_O with 6-min irradiation [no statistical significance according to one-way analysis of variance (ANOVA), *n* ≥ 3]. It is in accordance with the similar LSPR peak positions of AgNPs synthesized in different solvents (i.e., H_2_O and MOPS buffer). We also validated a positive correlation of NAD^+^-sensitized growth of AgNPs to the intensity of incident light and Ag^+^ ion concentration (fig. S7, F and G). On the other hand, we observed the broadening of the LSPR band in a MOPS buffer with the illumination time (Fig. 3A). It can be attributed to the faster growth of AgNPs in a MOPS buffer (than in H_2_O) and an increase in the SD of the AgNPs’ diameters from 4.8 to 6.1 nm after 6-min irradiation (fig. S7, E, H, and I). Note that the LSPR band position of an AgNP is dependent on the diameter of the metallic nanoparticle because its diameter determines the frequency of harmonic oscillation of surface electrons of the AgNP (*20*). In addition, we observed spectral shifts of the LSPR peak position from 450 to ca. 390 and 530 nm (Fig. 3A) along with the broadened size distribution of the AgNPs (fig. S7I). It suggests that the photochemical synthesis of AgNPs occurs through Ostwald ripening (*23*), in which Ag atoms are transferred from medium-sized particles to larger ones; thus, the populations of both smaller and larger particles increase simultaneously. On the basis of these results, we have depicted a plausible pathway of photoinduced electron transfer for the growth of AgNPs (Fig. 3, C and D). [NAD^+^]* transfers its photoexcited electrons to Ag^+^ ions in an oxidative quenching step or receives electrons from H_2_O (or MOPS) in a reductive quenching step. In a catalytic turnover step, the subsequent [NAD^+^]^•+^ or [NAD^+^]^•−^ drives H_2_O (or MOPS) oxidation or AgNPs formation, respectively, restoring the initial state of NAD^+^. Note that the formation of AgNPs is thermodynamically favorable because the reduction potential of Ag^+^ ions (0.59 V versus Ag/AgCl) is more positive than LUMO_Nicotinamide_ or LUMO_Adenine_. Overall, our results indicate that NAD^+^ has strong photocatalytic properties for metal ion reduction.

### Direct photoactivation of redox enzymes by NAD^+^

NAD^+^ is a prominent redox cofactor for activating numerous oxidoreductases [e.g., alcohol dehydrogenase (*24*), formate dehydrogenase (*25*), and xylitol dehydrogenase (*26*)] through hydride transfer. We found an alternative route for activating redox enzymes: NAD^+^ that functions not as a cofactor but as a photocatalyst, delivering its photoexcited electrons directly to the enzyme prosthetic group. This approach couples redox biocatalysis with photocatalysis, enabling nonphotocatalytic enzymes to perform photobiocatalytic redox reactions (*7*). As a model enzyme, we have used a flavin-containing OYE homolog from *Thermus scotoductus* (*Ts*OYE) that requires NADH as a cofactor for catalytic activities. The flavoenzyme reduces prosthetic flavin mononucleotide (FMN) to catalyze asymmetric trans-hydrogenation of activated C═C bonds; the catalyzed enantioselective hydrogenation was highlighted by the 2001 Nobel Prize in Chemistry (*27*).

**Fig. 4:**
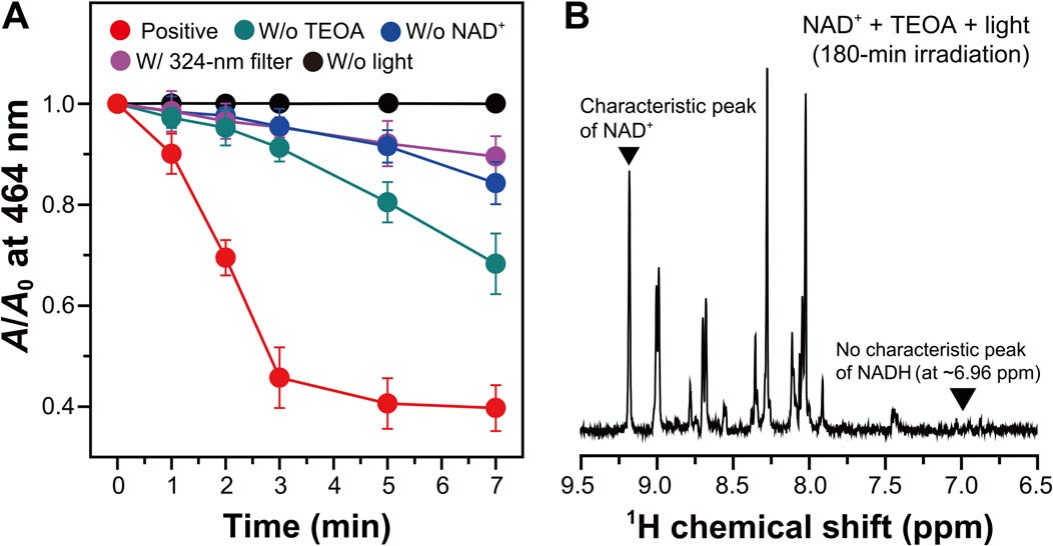
Direct photoactivation of *Ts*OYE-bound FMN using NAD^+^. (**A**) Changes in absorbance of *Ts*OYE-bound FMN with or without NAD^+^, TEOA, light, and 324-nm filter. Reaction conditions of the experimental group: 13.5 μM *Ts*OYE, 2 mM NAD^+^, and 5 mM CaCl_2_ in N_2_-purged TEOA buffer (100 mM and pH 7.5). A TEOA buffer was replaced by a MOPS buffer for a control experiment. Error bars correspond to the SD (*n* = 3). (**B**) ^1^H nuclear magnetic resonance (NMR) spectrum of a reaction sample consisting of 2 mM NAD^+^ in a TEOA buffer (100 mM, pH 7.5) under illumination [xenon lamp: λ, 260 to 900 nm; *P*_260–300 nm_, 0.023 μE cm^−2^ s^−1^ (10 mW cm^−2^); *P*_260–900 nm_, 0.970 μE cm^−2^ s^−1^ (200 mW cm^−2^)] for 180 min. ppm, parts per million.

We verified the reduction of the enzyme-bound FMN of *Ts*OYE by photoactivated NAD^+^ in a MOPS buffer using UV-Vis spectroscopy (see the rationale for using MOPS in the Materials and Method section). According to the literature (*28*), the reduction of the prosthetic FMN to FMNH^−^ (or FMNH_2_) through proton-coupled electron transfer causes an absorbance decrease at 464 nm. We observed these characteristic phenomena only under irradiation, and the presence of NAD^+^ decreased the absorbance more than the absence of NAD^+^ (fig. S8A). Because of the rather slow reduction of the prosthetic FMN, we substituted MOPS with triethanolamine (TEOA) to improve the rate of the overall photoinduced cascade of electron transfer. Note that TEOA is an extensively used electron donor in photocatalytic reduction reactions [e.g., H_2_ evolution (*29*) and cytochrome P450 activation (*30*)]. The use of TEOA augmented the decrease in the relative absorbance (*A*/*A*_0_) of prosthetic FMN by 46% for 3-min illumination compared to the use of MOPS (Fig. 4A). We attribute the result to the higher oxidation rate of TEOA than that of MOPS by photoactivated NAD^+^. On the other hand, *A*/*A*_0_ of *Ts*OYE-bound FMN decreased under irradiation without NAD^+^ (fig. S8B); the overall rate of the *A*/*A*_0_ decrease was lower, and the convergence value of *A*/*A*_0_ was higher than those in the presence of NAD^+^. We attribute the FMN’s absorbance decrease in the absence of NAD^+^ to direct photoreduction of FMN by TEOA. According to the literature (*7*), sacrificial electron donors can directly reduce flavin derivatives under illumination. In addition, *A*/*A*_0_ under the filtered light (324 nm < λ < 900 nm) was comparable to that in the absence of NAD^+^ (Fig. 4A), which is ascribed to the negligible photosensitization of NAD^+^.

The reduction of the prosthetic FMN in *Ts*OYE was not mediated by NADH through hydride transfer because photochemical activation of NAD^+^ does not generate its reduced form (i.e., NADH). We illuminated a TEOA-buffered solution of NAD^+^ and observed a negligible characteristic peak of NADH at 6.96 parts per million (*31*) in the ^1^H nuclear magnetic resonance (NMR) spectrum (Fig. 4B). Furthermore, the characteristic absorbance of NADH at 340 nm in the UV-Vis spectrum (*32*) was imperceptible (fig. S8C). Taking into account the spectroscopic analyses, we have depicted a possible pathway of electron transfer for direct activation of *Ts*OYE by photoexcited NAD^+^ in fig. S8 (D to F). Light promotes electrons of NAD^+^ from a ground state to an excited state; the photoexcited electrons have a potential energy enough to reduce a prosthetic FMN (−0.43 V versus Ag/AgCl). TEOA donates its electron (0.86 V versus Ag/AgCl) (*7*), reinstating the initial state of NAD^+^.

**Fig. 5:**
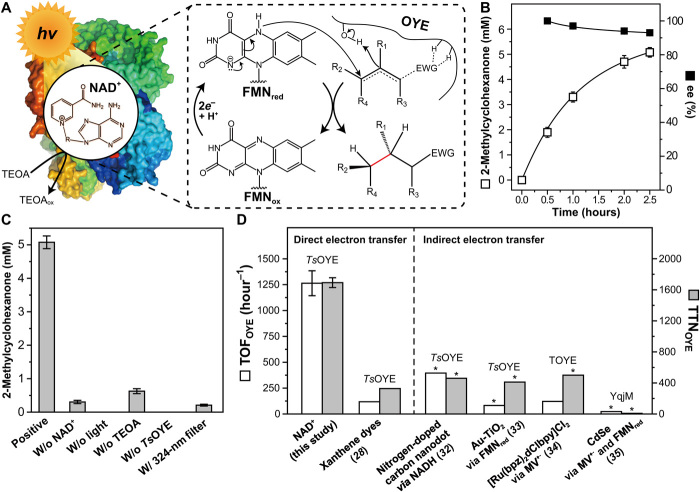
Hydrogenation of activated C═C bonds via direct, light-driven activation of *Ts*OYE using NAD^+^. (**A**) Photobiocatalytic reduction of activated C═C bonds by *Ts*OYE. Photoactivated NAD^+^ transfers its photoexcited electrons to the prosthetic FMN. A substrate is *trans*-hydrogenated by receiving a hydride from the reduced FMN and a proton from a Tyr residue. The oxidized NAD^+^ returns to its initial state after receiving electrons from electron donors. (**B**) Time profile of the photoenzymatic reduction of 2-methyl-2-cyclohexen-1-one and enantiomeric excess (ee) of the chiral product. (**C**) A series of deletional control experiments. Reaction conditions of the experimental group in (B) and (C): 1.5 mM NAD^+^, 3 μM *Ts*OYE, 5 mM CaCl_2_, and 6 mM substrate in a TEOA buffer (150 mM, pH 7.5) at 318.15 K. *P*_260–900 nm_, 1.212 μE cm^−2^ s^−1^ (250 mW cm^−2^). The measurement was performed in triplicate, and all reported values represent means ± SD. (**D**) Comparison of photobiocatalytic efficiencies for hydrogenation reaction driven by OYE. Asterisks (*) denote the approximate estimation according to data corroborated by the corresponding reference. MV, methyl viologen; TOYE, OYE from *Thermoanaerobacter pseudethanolicus* E39; YqjM, OYE from *Bacillus subtilis*.

The iterative delivery of photoexcited electrons from NAD^+^ to *Ts*OYE should execute a sustainable asymmetric hydrogenation of C═C bonds in α,β-unsaturated compounds (Fig. 5A). We tested 2-methyl-2-cyclohexen-1-one as an enone substrate because the tertiary carbon atom of the substrate becomes a chiral center after *Ts*OYE-catalyzed reduction. We observed a stereoselective conversion of 2-methyl-2-cyclohexen-1-one to 2-methylcyclohexanone (93 ± 1% enantiomeric excess) after 150-min illumination (Fig. 5B). The yield of the enantioenriched product was highest when the reaction occurred through the course of an electron cascade from TEOA to *Ts*OYE via photoexcited NAD^+^ (Fig. 5C), which is consistent with the highest reduction rate of the enzyme-bound FMN under these conditions (Fig. 4A). A turnover frequency of *Ts*OYE (TOF_*Ts*OYE_) and its total turnover number (TTN_*Ts*OYE_) were estimated to be 1263.4 ± 120.1 hour^−1^ and 1692.3 ± 63.3, respectively, with 1.5 mM NAD^+^ and 3 μM *Ts*OYE. These values are substantially higher than other reports on the combination of OYEs and photocatalysts (*28*, *32*–*35*) shown in Fig. 5D. The TTN of NAD^+^ (TTN_NAD+_) increased with the decreasing NAD^+^ concentration; it reached 168 with 9 μM *Ts*OYE (fig. S9A). Furthermore, the NAD^+^/*Ts*OYE hybrid exhibited a catalytic activity toward an unsaturated aldehyde (i.e., *trans*-cinnamaldehyde). The production yield of 3-phenylpropionaldehyde was lower than that of 2-methylcyclohexanone (fig. S9B), which is attributed to the lower specific activity of *Ts*OYE toward the aldehyde (*36*) and the inhibitory influence of the product on *Ts*OYE (*28*).

## DISCUSSION

The present work unveils the capability of NAD^+^ as a metal-free molecular photocatalyst. Through spectroscopic and electrochemical analyses, nicotinamide and adenine moieties of NAD^+^ absorb light (HOMO-LUMO gap, 4.13 eV) and consequently excite delocalized electrons in conjugated π bonds (LUMO_Nicotinamide_, −0.90 V; HOMO_Nicotinamide_, 3.23 V; LUMO_Adenine_, −2.89 V; HOMO_Adenine_, 1.24 V versus Ag/AgCl). On the basis of the HOMO and LUMO levels of NAD^+^ as thermodynamic indices, we have substantiated NAD^+^-sensitized reduction of O_2_ coupled with H_2_O oxidation. The capability of a photocatalyst to oxidize H_2_O as an electron donor is desirable in photochemical reduction reactions because of its abundance (i.e., 55 M in pure water) and no requirement of additional sacrificial electron suppliers. With H_2_O identified as an electron donor of photoactivated NAD^+^, we have demonstrated photocatalytic reduction of Ag^+^ ions to form AgNPs in deionized water, which are widely used for catalytic and biomedical applications [e.g., plasmonic oxidation reactions (*37*) and antibacterial activity (*38*)]. Furthermore, the use of MOPS as a sacrificial electron donor boosted the formation rate of AgNPs. This reduction experiment takes advantage of the cation-π interaction between Ag^+^ ions and aromatic moieties of NAD^+^.

Furthermore, this work reports the first example of NAD^+^-sensitized activation of redox enzymes through the reduction of the prosthetic groups, which is a departure from the traditional context of NADH regeneration coupled with enzymatic reactions. In the course of the NADH regeneration process, NAD^+^ is reduced to an enzymatically active form of NADH by an additional catalytic system (e.g., secondary enzyme or organometallic electron mediator) (*27*). Specifically, the photochemical regeneration of NADH requires a photocatalyst, an electron mediator, a sacrificial electron donor, and light (fig. S9C). Compared with the multicomponent regeneration method, the photobiocatalytic platform using NAD^+^ as a photocatalyst drastically simplifies the scheme by not using an electron mediator and a photocatalyst that reduces the electron mediator (fig. S9D). Besides its systematic simplicity, our direct photoactivation of OYE by NAD^+^ achieved the highest TOF_OYE_ and TTN_OYE_ ever recorded in photobiocatalytic transformation driven by OYE. We anticipate that this simple and efficient platform can enhance the enzymatic productivity through the molecular tuning of NAD^+^ in the future. Concurrently, the platform can vitalize the synthetic route for metal-free trans-hydrogenation, which is very rare in the production of pharmaceuticals and fine chemicals (*39*).

In conclusion, the current work identifies a new role of NAD^+^ beyond biological energy transduction. Under dark conditions, NAD^+^ itself cannot oxidize H_2_O and reduce O_2_ and metal ions. The oxidized form of natural cofactor cannot activate OYEs for biocatalytic transformation because the prosthetic group requires a hydride ion from a redox cofactor. This nonproductive property of NAD^+^ is overturned by shining light on NAD^+^; it can function as a photocatalyst to reduce O_2_, to oxidize H_2_O, to grow metallic nanoparticles, and to directly activate redox enzymes for solar-to-chemical conversion.

## MATERIALS AND METHODS

### Chemicals

β-NAD^+^ hydrate (NAD^+^), nicotinamide, adenine, d-(−)-ribose, deuterium oxide (D_2_O), tetrabutylammonium hexafluorophosphate (TBAPF_6_), acetonitrile, sodium phosphate monobasic, sodium phosphate dibasic, ferrocene, NBT, Tris, acetic acid, acetylacetone, ammonium acetate, MOPS, AgNO_3_, TEOA, calcium chloride (CaCl_2_), ethyl acetate, magnesium sulfate (MgSO_4_), 1-octanol, 2-methyl-2-cyclohexen-1-one, *trans*-cinnamaldehyde, and 3-phenylpropionaldehyde were purchased from Sigma-Aldrich (St. Louis, MO, USA) and used without further purification. *Ts*OYE was produced following a literature procedure reported previously (*32*).

### Spectroscopic analysis

UV-Vis spectra were recorded on a V-650 UV-Vis absorption spectrophotometer (JASCO Inc., Japan) using a quartz glass cuvette (path length, 1 cm). A ^1^H NMR spectrum was obtained using a 400-MHz and 54-mm NMR DD2 instrument (Agilent Technologies, USA) at 298.15 K. Note that an aqueous reaction sample was dissolved in D_2_O, and a water suppression technique was used to improve the signal-to-noise ratio.

### Electrochemical characterizations

All electrochemical experiments were performed on a potentiostat/galvanostat (WMPG 1000, WonATech Co., Korea). A three-electrode setup was used with a glassy carbon disk electrode (working electrode: electrode diameter, 3 mm), Ag/AgCl electrode (reference electrode; 3 M NaCl), and a platinum wire (counter electrode) in a single cell. The glassy carbon disk electrode was always polished using 1, 0.3, and 0.05 μm of deagglomerated alumina suspensions before electrochemical analysis. On the basis of the following equations (Eqs. 4 and 5) (*40*), we measured the formal potential of ferrocene and onset potentials of NAD^+^ to estimate LUMO and HOMO energy levels of NAD^+^

$$E_{\text{LUMO}}=-(E_{\text{onset},\text{red}}-E_{\text{formal}}+5.06)\text{eV}$$ (4)

$$E_{\text{HOMO}}=-(E_{\text{onset},\text{ox}}-E_{\text{formal}}+5.06)\text{eV}$$ (5)

Note that *E*_onset,red_ is the onset potential of NAD^+^ reduction, *E*_onset,ox_ is that of NAD^+^ oxidation, and *E*_formal_ is the formal potential of ferrocene. The electrolyte solution consisted of acetonitrile (containing 100 mM TBAPF_6_)/sodium phosphate buffer (100 mM, pH 7.5) (v/v, 1:1).

### NBT assay

To confirm the photochemical formation of O_2_^•−^, NAD^+^ and 30 μM NBT were dissolved in a phosphate buffer (50 mM, pH 7.5). We injected 500 μl of reaction sample into a 1.5-ml Eppendorf tube (SPL Life Sciences Co., Korea). The reaction volume and the vessel type in control groups were identical to those in the experimental group. The tube was irradiated by a xenon lamp (Newport Co., USA) equipped with an infrared water filter. After irradiation, we used a V-650 UV-Vis absorption spectrophotometer (JASCO Inc., Japan) to monitor a change in the absorbance at 560 nm. Note that NBT formazan, which forms by the reaction between O_2_^•−^ and NBT, exhibits a maximum absorption at 560 nm. The O_2_- or N_2_-rich solution was prepared by purging with O_2_ or N_2_, respectively, for 1 hour.

### Analysis of hydroxyl radical

To estimate the amount of OH^•^ produced by photoactivated NAD^+^, NAD^+^ and 10 mM Tris were dissolved in a sodium phosphate buffer (50 mM, pH 7.5). The solution was exposed to light (λ, 260 to 900 nm; *P*_260–900 nm_, 200 mW cm^−2^; *P*_260–300 nm_, 10 mW cm^−2^) from a xenon lamp (Newport Co., USA) equipped with an infrared filter at 293.15 K. The sample was then mixed with Nash’s reagent (v/v, 1:1); the reagent was composed of 50 μM acetic acid, 20 mM acetylacetone, and 2 M ammonium acetate. The incubation of the mixture at 323.15 K for 1 hour developed a yellow color, which was measured spectrophotometrically at 412 nm. We purged O_2_ or N_2_ gas into a reaction medium for 1 hour for an O_2_- or N_2_-enriched environment, respectively.

### Photoreduction of Ag^+^ ions to AgNPs

For photochemical formation of AgNPs, NAD^+^ and AgNO_3_ were dissolved in a MOPS buffer (50 mM, pH 7.5) and irradiated with a xenon lamp (Newport Co., USA) equipped with a water filter at 293.15 K. In this experiment, a sodium phosphate buffer was not used because silver phosphate precipitates (Ag_3_PO_4_) form by the reaction between silver ions and phosphate ions. An LSPR band of AgNPs was monitored using a V-650 UV-Vis absorption spectrophotometer (JASCO Inc., Japan). Before obtaining UV-Vis absorption spectra, the samples in a MOPS buffer were diluted 10-fold, whereas those in water were not diluted because the formation rate of AgNPs in a MOPS buffer was much higher than that in water. We observed AgNPs using a JEM 3010 transmission electron microscope (JEOL Co., Japan) at 300 kV. The quantification of AgNPs was conducted using an inductively coupled plasma mass spectrometer (7700x, Agilent Technologies, USA). Before mass spectrometric analysis, a reaction sample was put in a dialysis tubing (molecular weight cutoff, 500 to 1000) against deionized water for 24 hours to eliminate Ag^+^ ions in the sample. AgNPs were also quantified using a microbalance after their purification by centrifugation. We synthesized AgNPs in an Eppendorf tube, the mass of which was measured using a microbalance. After the photochemical reaction, the tube was centrifuged at 27,237*g* for 25 min, making AgNPs concentrated as a dark pellet. The supernatant was discarded as much as possible, and deionized water was added to the tube. This washing process was repeated five times, but deionized water was not added at the last repetition. The residual water was evaporated in a vacuum chamber for 10 hours, and the mass of the Eppendorf tube containing AgNPs was measured using the microbalance to calculate the mass of AgNPs.

### Photoenzymatic reaction and analysis

A TEOA-buffered solution (150 mM, pH 7.5) containing NAD^+^, *Ts*OYE, CaCl_2_, and substrate was prepared in a microcentrifuge tube. For the control experiment, in the absence of TEOA, MOPS was used as a substitute for TEOA because it is extensively used in the OYE biocatalysis, acts as an electron donor of photoactivated NAD^+^, and does not produce a precipitate with Ca^2+^ ions; the divalent ion is required for activity of *Ts*OYE (*41*). Note that MOPS and TEOA have buffering capacities. The sample was irradiated with a 450-W xenon lamp at 318.15 K. For the unit conversion of light intensity from mW cm^−2^ to μE cm^−2^ s^−1^, the average photon energy was ca. 2.14 eV according to the spectral irradiance of the xenon lamp. Note that μE cm^−2^ s^−1^ refers to the number of moles of photons in micromole hitting a defined surface per second. For quantitative analysis of the product using gas chromatography (GC), organic substrates and products were extracted with ethyl acetate solvent containing 1-octanol as an internal standard. The mixture was centrifuged to collect the organic phase and, after which, was dried with MgSO_4_ to eliminate residual water content. The organic supernatant was analyzed by GC using a 7890A gas chromatograph (Agilent Technologies, USA). The machine was equipped with a flame ionization detector and a CP-Chirasil-Dex CB column (25 m by 0.32 mm by 0.25 μm). The oven temperature program for all enzymatic substrates and products was 363.15 K held for 2 min, 4 K min^−1^ to 388.15 K held for 0 min, and 20 K min^−1^ to 453.15 K held for 1 min. The yield, enantiomeric excess (ee), TOF, and TTN were calculated according to the following equations (Eqs. 6 to 10)

$$\text{Yield}(\%)=\frac{\text{Concentration of product}}{\text{Initial concentration of substrate}}\times 100$$ (6)

$$\text{ee}(\%)=\frac{|\text{Moles of an enantiomer}-\text{Moles of the other enantiomer}|}{\text{Total moles of product}}\times 100$$ (7)

$${\text{TOF}}_{\mathrm{Ts}\text{OYE}}({\text{hour}}^{-1})=\frac{\text{Concentration of product at the given time}}{\text{Concentration of}\;\mathrm{Ts}\text{OYE}\times \text{Time}}$$ (8)

$${\text{TTN}}_{\mathrm{Ts}\text{OYE}}=\frac{\text{Maximum concentration of product}}{\text{Concentration of}\;\mathrm{Ts}\text{OYE}}$$ (9)

$${\text{TTN}}_{\text{NAD}+}=\frac{\text{Maximum concentration of product}}{\text{Concentration of}\;{\text{NAD}}^{+}}$$ (10)

## Supplementary Material

http://advances.sciencemag.org/cgi/content/full/5/7/eaax0501/DC1

Download PDF

Nicotinamide adenine dinucleotide as a photocatalyst

## SUPPLEMENTARY MATERIALS

Supplementary material for this article is available at http://advances.sciencemag.org/cgi/content/full/5/7/eaax0501/DC1 Fig. S1. Optical and electrochemical properties of NAD^+^. Fig. S2. Photostability of NAD^+^. Fig. S3. Formation of superoxide radicals by photoactivated NAD^+^. Fig. S4. Use of Tris and Nash’s reagent in quantification of hydroxyl radicals. Fig. S5. Solar-driven formation of hydroxyl radicals with NAD^+^. Fig. S6. NAD^+^-sensitized production of AgNPs without sacrificial electron donors. Fig. S7. Photocatalytic synthesis of AgNPs using NAD^+^ in a MOPS buffer. Fig. S8. Photoreduction of prosthetic FMN driven by NAD^+^. Fig. S9. Light-driven enzymatic hydrogenation of C═C bonds using NAD^+^ and *Ts*OYE.
