# Supplementary material for: Nicotinamide adenine dinucleotide as a photocatalyst
Source: Sci Adv. 2019 Jul 19;5(7):eaax0501. doi: 10.1126/sciadv.aax0501 (PMC6641943; doi:10.1126/sciadv.aax0501)
Supplement: http://advances.sciencemag.org/cgi/content/full/5/7/eaax0501/DC1 [file supp_5_7_eaax0501__index.html]

Science Advances | Science AdvancesAAASSearchScience AdvancesMenu

## Supplementary Materials

**This PDF file includes:**

- Fig. S1. Optical and electrochemical properties of NAD+.
- Fig. S2. Photostability of NAD+.
- Fig. S3. Formation of superoxide radicals by photoactivated NAD+.
- Fig. S4. Use of Tris and Nash’s reagent in quantification of hydroxyl radicals.
- Fig. S5. Solar-driven formation of hydroxyl radicals with NAD+.
- Fig. S6. NAD+-sensitized production of AgNPs without sacrificial electron donors.
- Fig. S7. Photocatalytic synthesis of AgNPs using NAD+ in a MOPS buffer.
- Fig. S8. Photoreduction of prosthetic FMN driven by NAD+.
- Fig. S9. Light-driven enzymatic hydrogenation of C═C bonds using NAD+ and *Ts*OYE.

Download PDF

**Files in this Data Supplement:**

- Adobe PDF - aax0501\_SM.pdf
