# Supplementary material for: Nicotinamide adenine dinucleotide as a photocatalyst
Source: Sci Adv. 2019 Jul 19;5(7):eaax0501. doi: 10.1126/sciadv.aax0501 (PMC6641943; doi:10.1126/sciadv.aax0501)
Supplement: Download PDF [file aax0501_SM.pdf]

Science Advances
AAAS

advances.sciencemag.org/cgi/content/full/5/7/eaax0501/DC1

Supplementary Materials for

Nicotinamide adenine dinucleotide as a photocatalyst

Jinhyun Kim, Sahng Ha Lee, Florian Tieves, Caroline E. Paul, Frank Hollmann, Chan Beum Park*

*Corresponding author. Email: parkcb@kaist.ac.kr

Published 19 July 2019, *Sci. Adv.* **5**, eaax0501 (2019)
DOI: 10.1126/sciadv.aax0501

This PDF file includes:

Fig. S1. Optical and electrochemical properties of NAD^+.
Fig. S2. Photostability of NAD^+.
Fig. S3. Formation of superoxide radicals by photoactivated NAD^+.
Fig. S4. Use of Tris and Nash's reagent in quantification of hydroxyl radicals.
Fig. S5. Solar-driven formation of hydroxyl radicals with NAD^+.
Fig. S6. NAD^+-sensitized production of AgNPs without sacrificial electron donors.
Fig. S7. Photocatalytic synthesis of AgNPs using NAD^+ in a MOPS buffer.
Fig. S8. Photoreduction of prosthetic FMN driven by NAD^+.
Fig. S9. Light-driven enzymatic hydrogenation of C═C bonds using NAD^+ and *Ts*OYE.

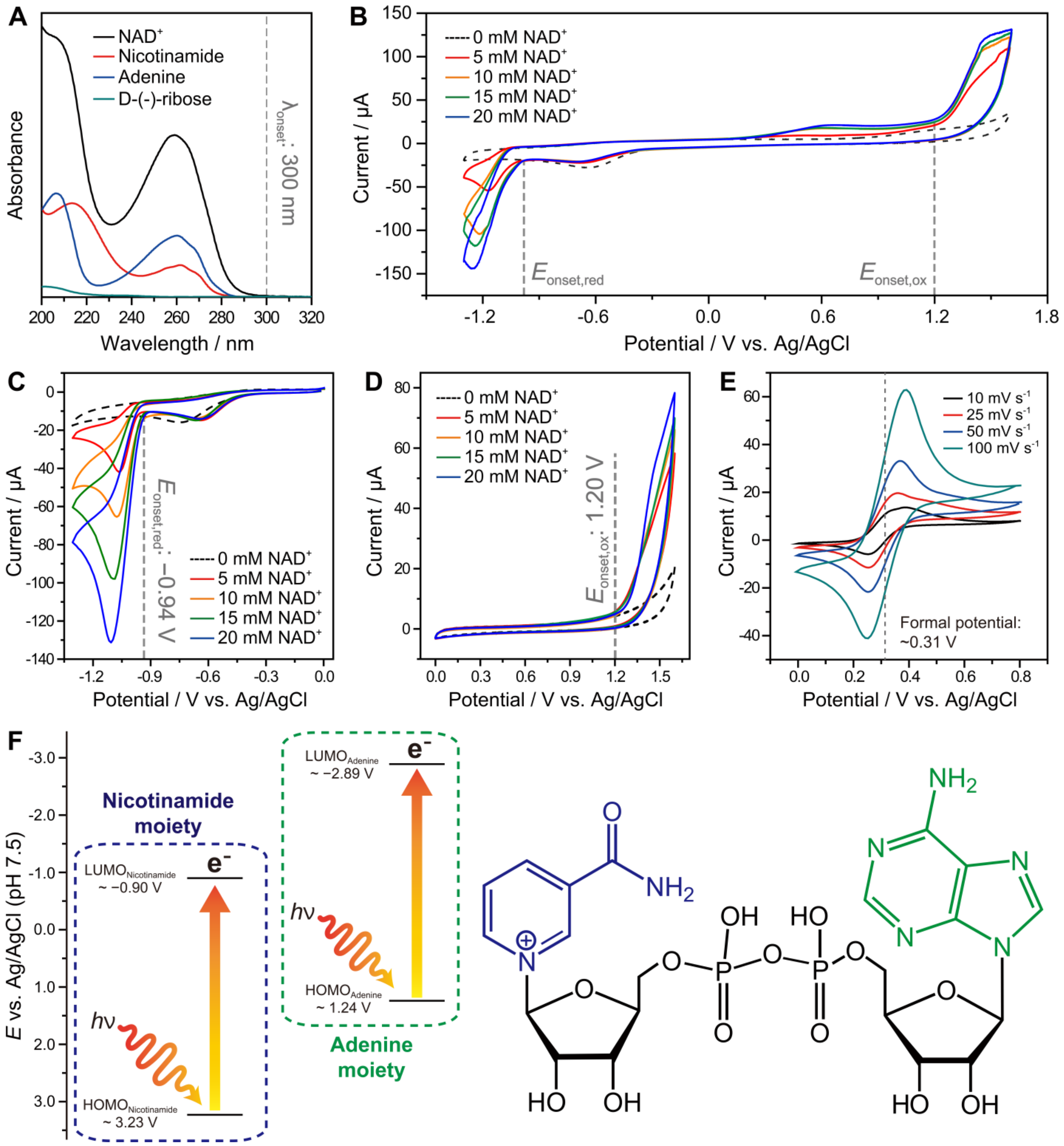

Fig. S1. Optical and electrochemical properties of NAD$^+$. (**A**) UV-Vis absorption spectra of 50 µM NAD$^+$, 50 µM nicotinamide, 50 µM adenine, and 50 µM D-(-)-ribose in deionized water. Cuvette path length: 1 cm. (**B, C, D**) Cyclic voltammograms of NAD$^+$ with different potential ranges. Working electrode: a polished glassy carbon electrode. Scan rate: 100 mV s^{-1}. (**E**) Cyclic voltammograms of 5.38 mM ferrocene at different scan rates. Electrolyte solution of (**B, C, D, E**): 1/1 mixture of acetonitrile (containing 100 mM $TBAPF_6$)/sodium phosphate buffer (100 mM, pH 7.5). The number of cycles of (**B, C, D, E**): 4. (**F**) Energy levels of nicotinamide and adenine moieties of NAD$^+$ (Left). Molecular structure of NAD$^+$ (Right).

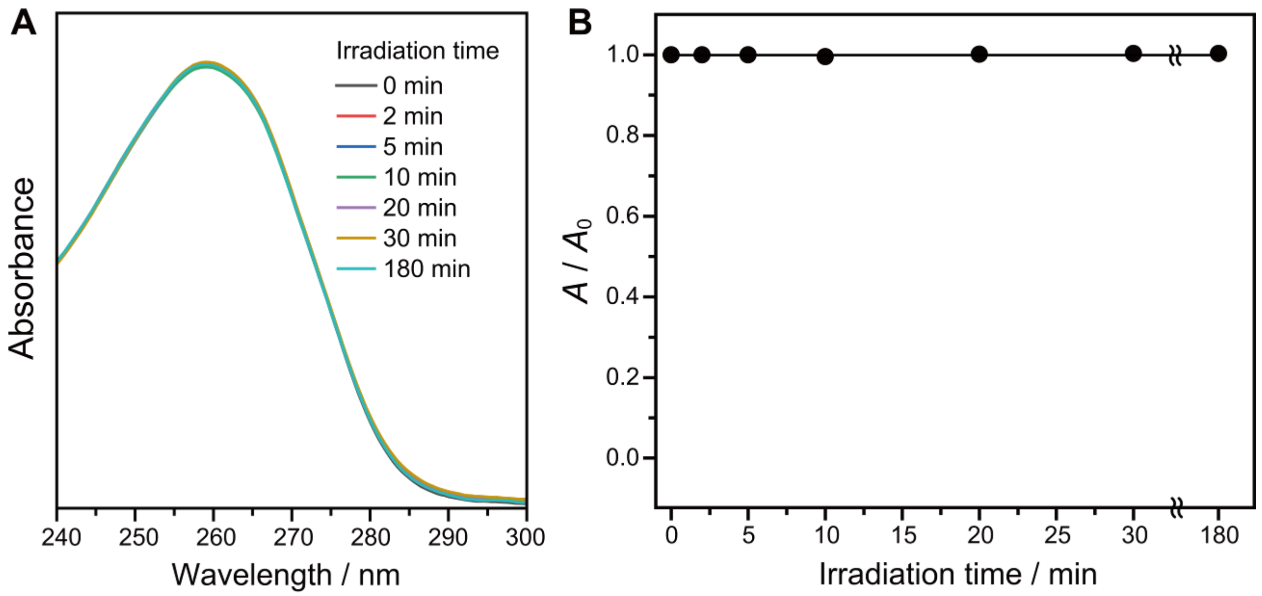

Fig. S2. Photostability of NAD^+. (A) Spectrophotometric changes in the absorbance of NAD^+ under irradiation (λ: 260-900 nm, $P_{260\text{-}900\ nm}$: 200 mW cm^{-2}, $P_{260\text{-}300\ nm}$: 10 mW cm^{-2}) at 293.15 K. **(B)** Changes in relative absorbance (A/A_0) of NAD^+ at 260 nm. Solvent: deionized water. Light source: a xenon lamp equipped with a water filter.

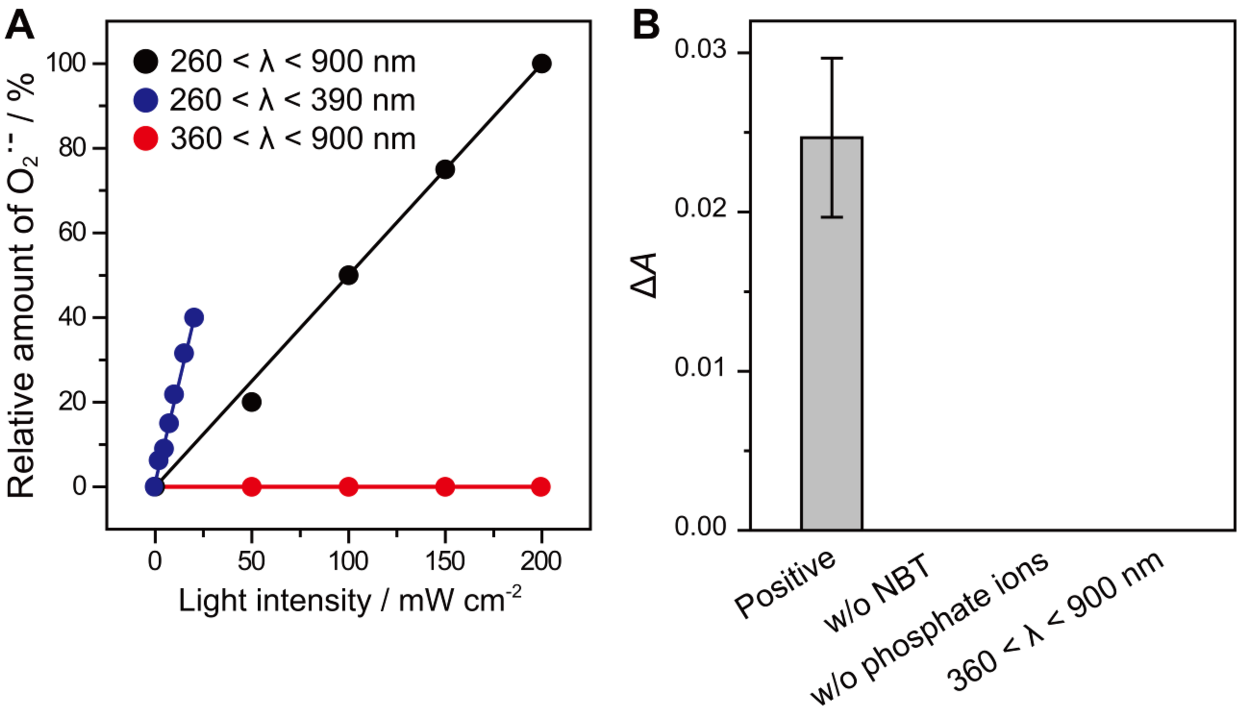

Fig. S3. Formation of superoxide radicals by photoactivated NAD^+. (A) Influence of wavelength range and light intensity on the light-driven formation of superoxide radicals. A buffered solution containing 1 mM NAD^+ and 30 μM NBT was irradiated with a xenon lamp with/without a filter for 30 min. Solvent: a sodium phosphate buffer (50 mM, pH 7.5, O_2 purged). A relative amount of the radical is the normalized absorption intensity (at a given wavelength range and light intensity) to that at $P_{260\text{-}900\ nm}$ of 200 mW cm^{-2}. (**B**) Control experiments for a background reaction with NBT and phosphate ions under UV illumination. Reaction condition of the experimental group: 30 μM NBT, O_2-purged sodium phosphate buffer (50 mM, pH 7.5), irradiation (Xenon lamp, 200 mW cm^{-2}, 30 min). The measurement was performed in triplicate and all reported values represent the mean ± standard deviation.

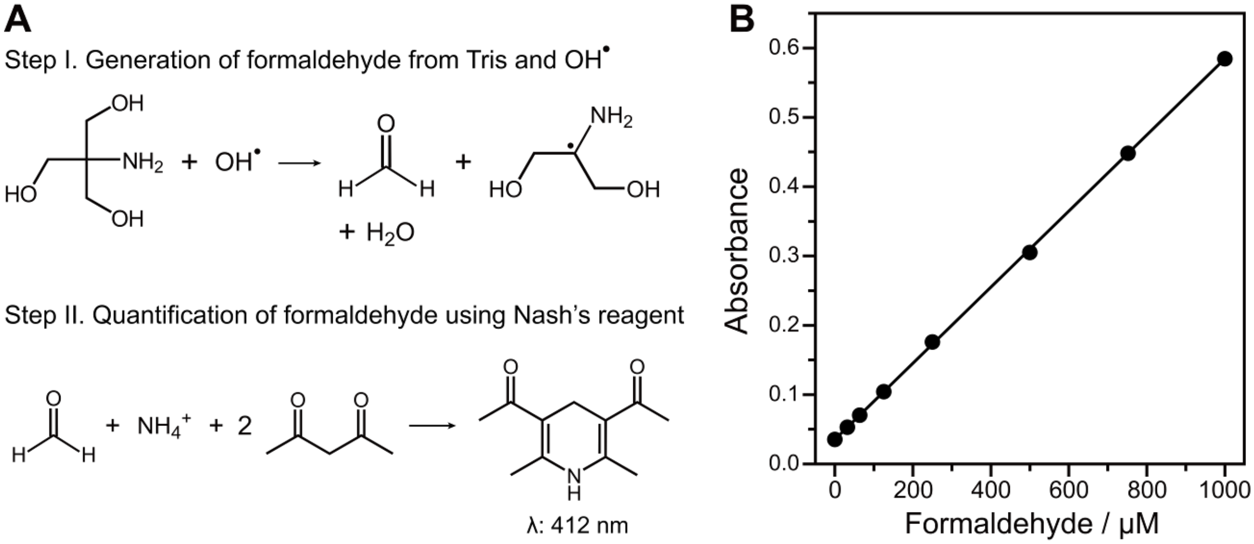

Fig. S4. Use of Tris and Nash's reagent in quantification of hydroxyl radicals. (A) Reaction between a Tris and a hydroxyl radical generates a formaldehyde molecule; one molecule of formaldehyde reacts with one ammonium ion and two molecules of acetylacetone to produce 1-(5-acetyl-2,6-dimethyl-1,4-dihydropyridin-3-yl)ethanone. The concentration of the final product can be quantitatively measured at 412 nm. (**B**) Calibration of formaldehyde using Nash's reagent. Absorbance at 412 nm was recorded for 0-1 mM formaldehyde after the incubation of a solution containing formaldehyde and Nash's reagent at 323.15 K for 1 h.

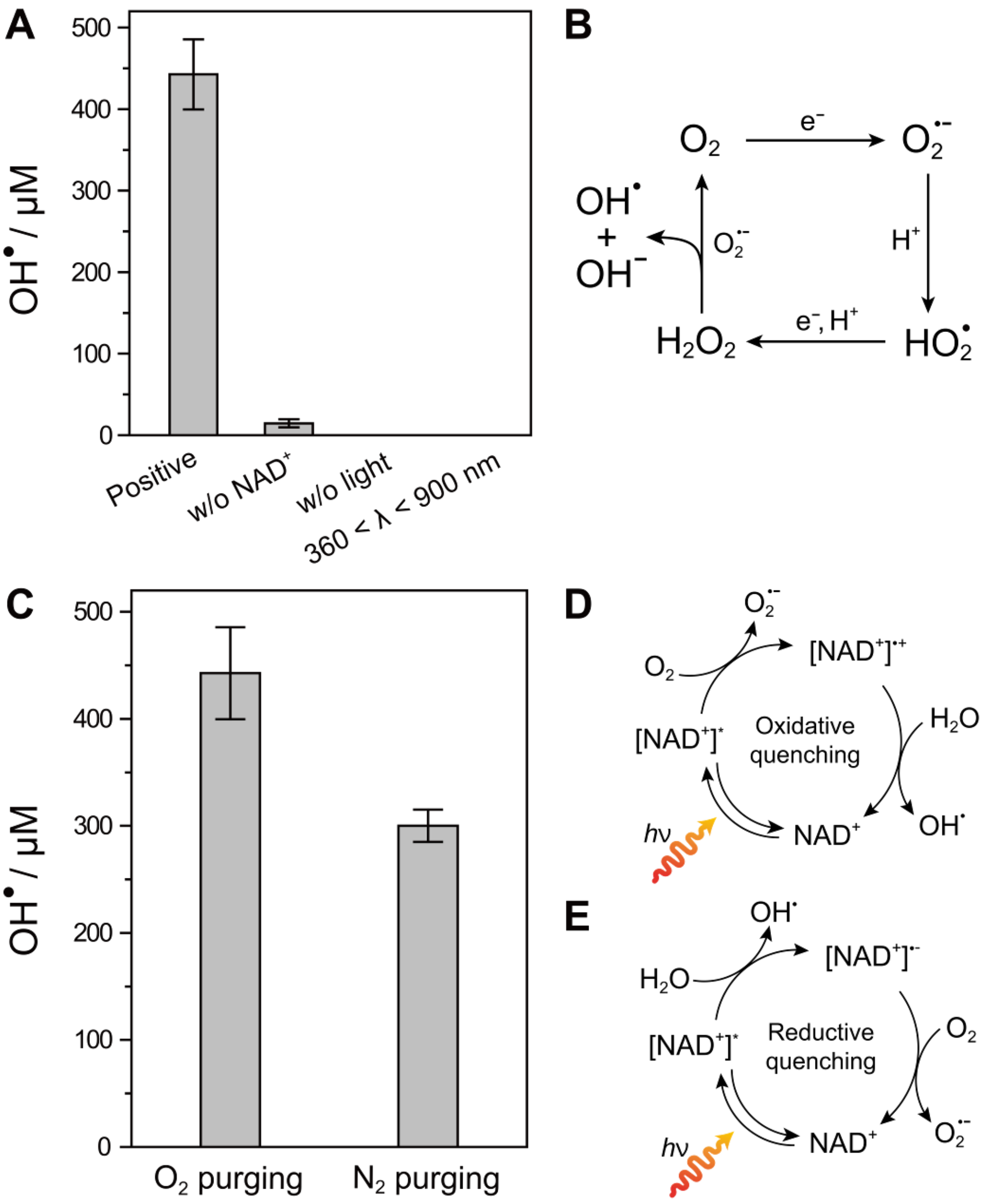

Fig. S5. Solar-driven formation of hydroxyl radicals with NAD$^+$. (A) Control experiments for NAD$^+$-sensitized formation of hydroxyl radicals. Reaction condition of the experimental group: 1 mM NAD$^+$ and 10 mM Tris in an O_2-purged sodium phosphate buffer (50 mM, pH 7.5) under light condition (λ: 260-900 nm, $P_{260\text{-}300\ nm}$: 10 mW cm^{-2}, $P_{260\text{-}900\ nm}$: 200 mW cm^{-2}, t = 30 min). Temperature: 293.15 K. (**B**) Mechanism of OH$^\bullet$ generation in the process of O_2 reduction. Hydroxyl radicals can be formed via the reaction between H_2O_2 and $O_2^{\bullet-}$. (**C**) Effect of the N_2-rich condition on the generation of hydroxyl radicals by NAD$^+$ photocatalyst. Reaction condition: 1 mM NAD$^+$ and 10 mM Tris in a sodium phosphate buffer (50 mM, pH 7.5) under irradiation (λ: 260-900 nm, $P_{260\text{-}300\ nm}$: 10 mW cm^{-2}, $P_{260\text{-}900\ nm}$: 200 mW cm^{-2}, t = 30 min) at 293.15 K. Error bars correspond to the standard deviation (n = 3). (**D, E**) Two possible photocatalytic pathways of NAD$^+$-driven O_2 reduction and H_2O oxidation. (**D**) Oxidative quenching step. (**E**) Reductive quenching step.

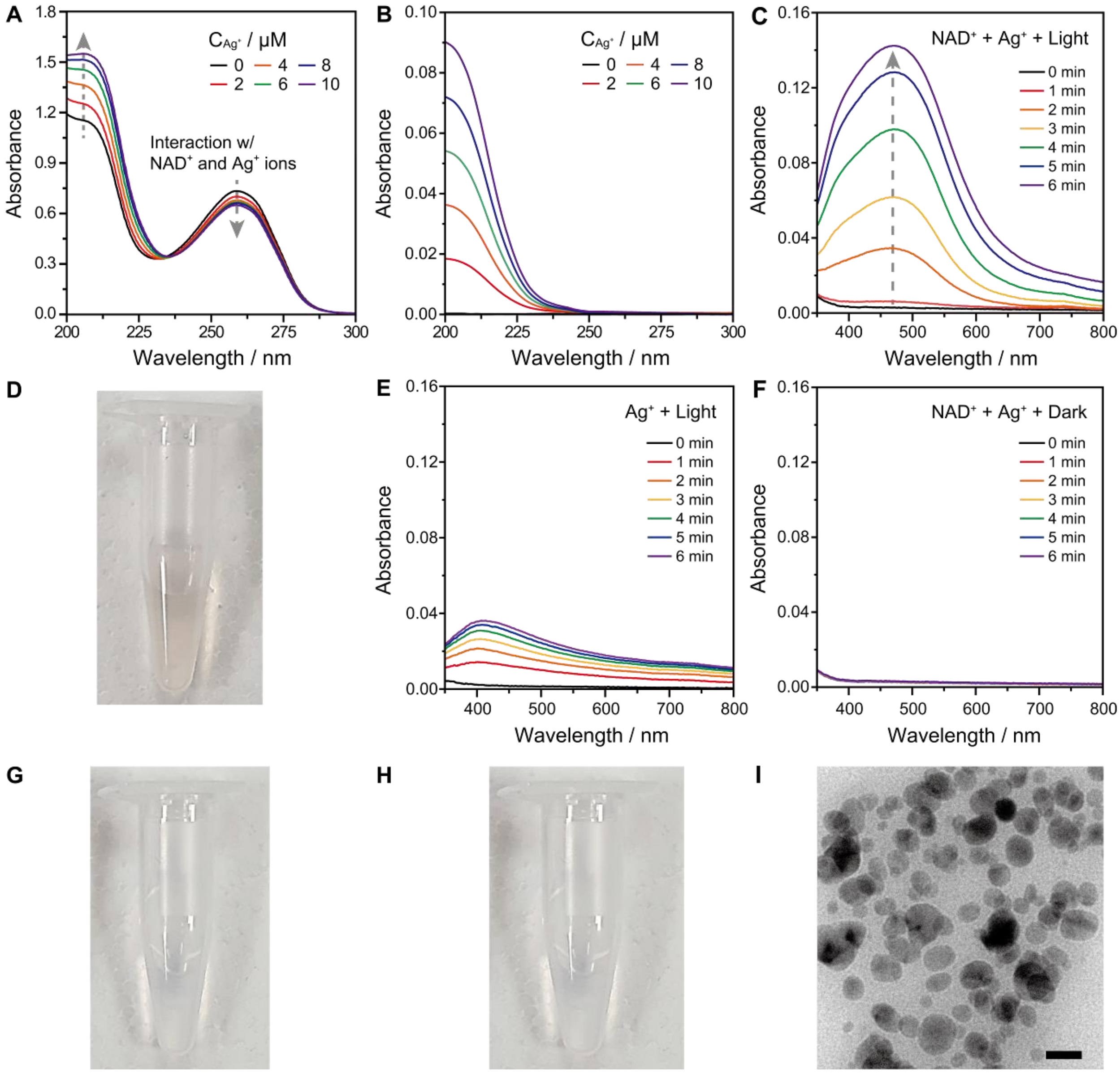

Fig. S6. NAD$^+$-sensitized production of AgNPs without sacrificial electron donors. (A) Spectrophotometric changes in the absorbance of 50 μM NAD$^+$ upon addition of Ag$^+$ ions at 293.15 K. **(B)** UV-Vis absorption spectra of $AgNO_3$ (0-10 μM). (**C**) Changes in absorption spectra of NAD$^+$/Ag$^+$ mixture under illumination. Reaction condition: 0.5 mM NAD$^+$ and 1 mM $AgNO_3$ dissolved in a N_2-purged deionized water. Light source: a xenon lamp equipped with an infrared filter (λ: 260-900 nm, $P_{260\text{-}300\ nm}$: 5 mW cm^{-2}, $P_{260\text{-}900\ nm}$: 100 mW cm^{-2}). (**D**) Photograph of the experimental group after 6-min illumination. (**E, F**) Changes in absorption spectra without (**E**) NAD$^+$ or (**F**) light. (**G, H**) Photographs of control groups in the absence of (**G**) NAD$^+$ or (**H**) light after 6-min reaction. (**I**) High resolution-transmission electron microscopy image of AgNPs shown in (**C**) under 6-min irradiation. Scale bar: 20 nm. Photo credit: Jinhyun Kim, Korea Advanced Institute of Science and Technology.

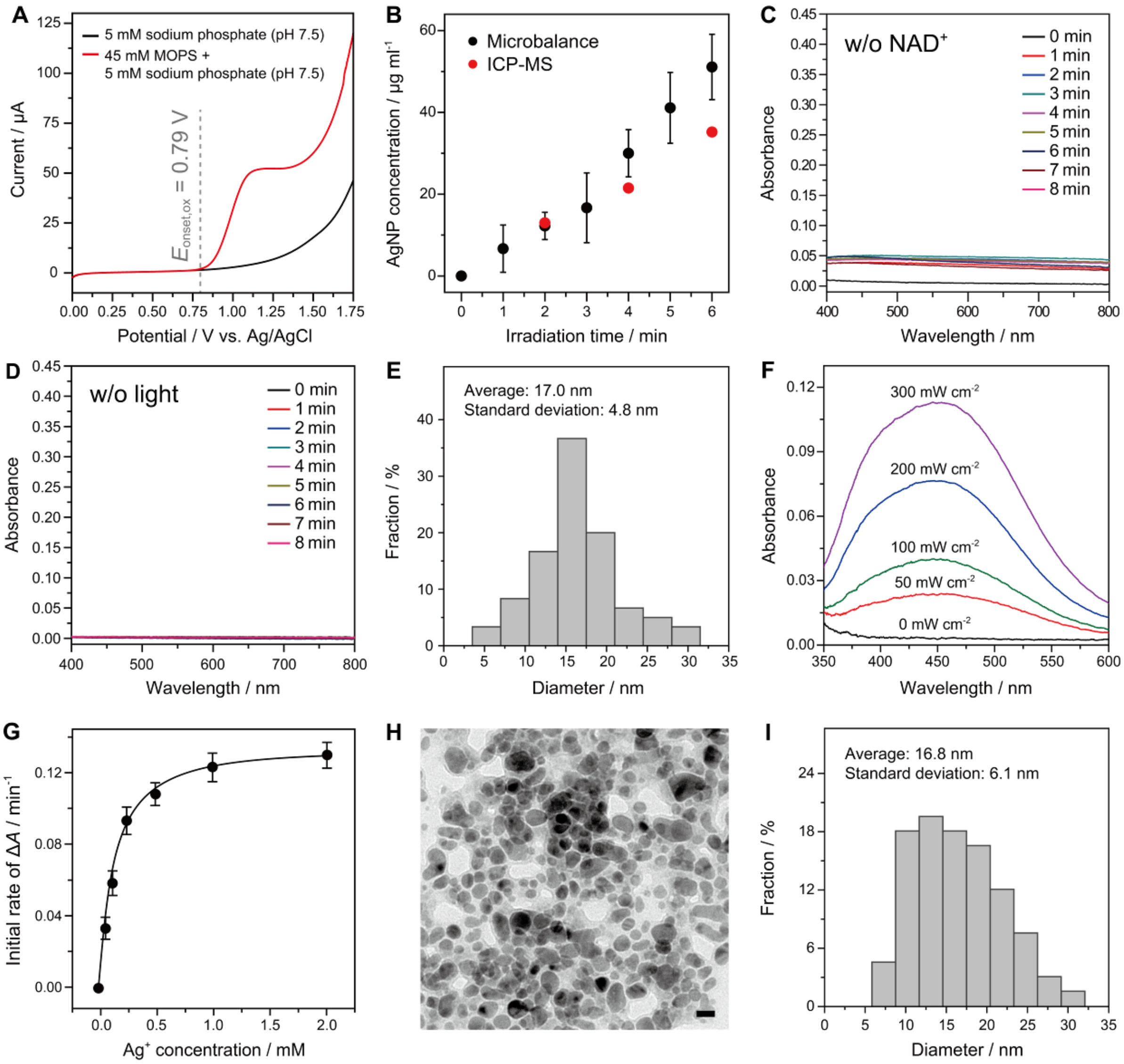

Fig. S7. Photocatalytic synthesis of AgNPs using NAD$^+$ in a MOPS buffer. (A) Linear sweep voltammogram of MOPS. Working electrode: a polished glassy carbon electrode. Counter electrode: a Pt wire. Scan rate: 25 mV s^{-1}. (**B**) Quantification of AgNPs (shown in **Fig. 3A**) using an inductively coupled plasma mass spectrometer (ICP-MS) or a microbalance. (**C, D**) Control experiments of AgNP formation by photoexcited NAD$^+$ without (**C**) NAD$^+$ or (**D**) light. Experimental experiment: **Fig. 3A**. (**E**) Size distribution histogram of AgNPs formed by photoexcited NAD$^+$ under 1-min irradiation. (**F**) Influence of light intensity on NAD$^+$-sensitized formation of AgNPs. Reaction condition: 0.25 mM NAD$^+$, 1 mM $AgNO_3$, N_2-purged MOPS buffer (50 mM, pH 7.5), irradiation ($P_{260\text{-}900\ nm}$: 100 mW cm^{-2}, t = 30 s), 293.15 K. (**G**) Effect of Ag$^+$ concentration on NAD$^+$-sensitized growth of AgNPs. Reaction condition: 10 µM NAD$^+$, $AgNO_3$, N_2-purged MOPS buffer (50 mM, pH 7. 5), irradiation ($P_{260\text{-}900\ nm}$: 100 mW cm^{-2}), 293.15 K. $\Delta A \equiv A(t = 30\ s) - A(t = 0\ s)$ at 442 nm. An error bar represents a standard deviation from three independent experiments. (**H**) High resolution-transmission electron microscopy image of AgNPs synthesized by photoactivated NAD$^+$ under 6-min illumination. Scale bar: 20 nm. (**I**) Size distribution histogram of AgNPs shown in (**H**).

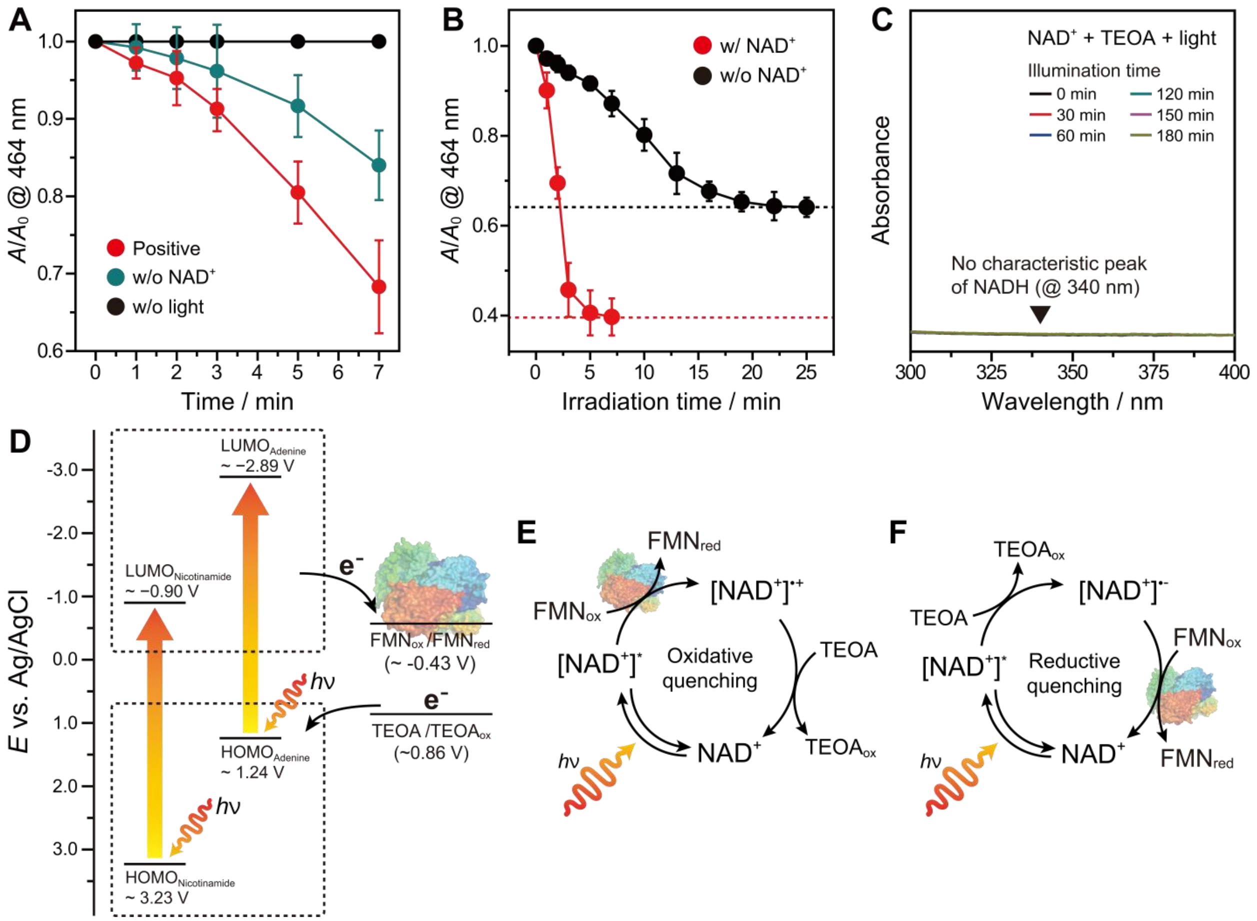

Fig. S8. Photoreduction of prosthetic FMN driven by NAD^+. (A) NAD^+-sensitized reduction of FMN bound to *Ts*OYE in a MOPS buffer. Reaction condition of the experimental group: 13.5 μM *Ts*OYE, 2 mM NAD^+, and 5 mM $CaCl_2$ in N_2-purged MOPS buffer (100 mM, pH 7.5). $P_{260\text{-}900\ nm}$: 0.485 μE cm^{-2} s^{-1}. (**B**) Light-driven activation of prosthetic FMN with/without NAD^+ in a TEOA buffer. Reaction condition of the experimental group: 13.5 μM *Ts*OYE, 2 mM NAD^+, and 5 mM $CaCl_2$ in N_2-purged TEOA buffer (100 mM, pH 7.5). $P_{260\text{-}900\ nm}$: 0.485 μE cm^{-2} s^{-1}. Error bars of (**A**) and (**B**): standard deviation (n = 3). (**C**) Negligible formation of NADH from NAD^+. Absorption spectra of 0.1 mM NAD^+ in a TEOA buffer (5 mM, pH 7.5) under illumination (Xenon lamp, λ: 260-900 nm, $P_{260\text{-}900\ nm}$: 0.970 μE cm^{-2} s^{-1}). (**D**) Energy diagram for prosthetic FMN reduction by photoactivated NAD^+. (**E, F**) Plausible mechanism of photoinduced reduction of prosthetic FMN by NAD^+ through (**E**) an oxidative quenching step or (**F**) a reductive quenching step. Photosensitization of NAD^+ excites its electrons from a ground state to the excited state. The photoexcited electrons possess a potential energy high enough to reduce the FMN prosthetic group. As a counterpart of the photocatalytic reduction, TEOA donates its electron to the oxidized NAD^+.

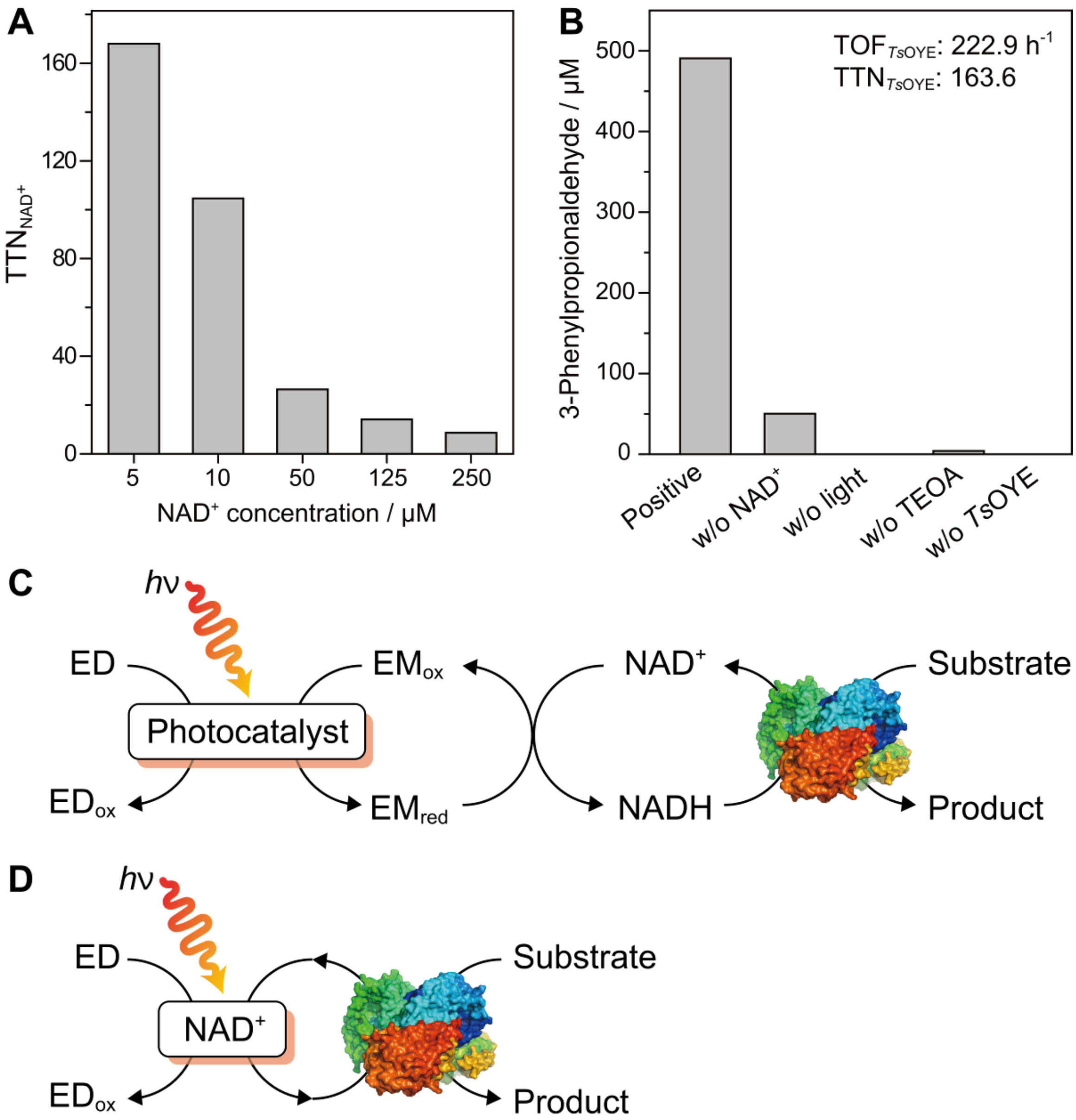

Fig. S9. Light-driven enzymatic hydrogenation of C=C bonds using NAD$^+$ and *Ts*OYE. (A) TTN of NAD$^+$ in photoenzymatic reduction by *Ts*OYE. Reaction conditions: NAD$^+$, 9 µM *Ts*OYE, 5 mM $CaCl_2$, and 6 mM 2-methyl-2-cyclohexen-1-one in a TEOA buffer (150 mM, pH 7.5) at 318.15 K. $P_{260\text{-}900\ nm}$: 1.212 µE cm^{-2} s^{-1}. TTN$_{NAD+}$s were determined after 150 min of reaction. (**B**) A series of control experiments for each reaction components (i.e., NAD$^+$, light, TEOA, *Ts*OYE). Reaction condition of the experimental group: 1.5 mM NAD$^+$, 3 µM *Ts*OYE, 5 mM $CaCl_2$, and 6 mM *trans*-cinnamaldehyde in a TEOA buffer (150 mM, pH 7.5) at 318.15 K. $P_{260\text{-}900\ nm}$: 1.212 µE cm^{-2} s^{-1}. TOF and TTN were determined after 30 and 120 min of reaction. The specific specific of *Ts*OYE towards *trans*-cinnamaldehyde (1.16 µmol min^{-1} mg^{-1}) is 24-times lower than that towards 2-methyl-2-cyclohexen-1-one (27.59 µmol min^{-1} mg^{-1}) [*Biochem. Biophys. Res. Commun.* **393**, 426-431 (2010)]. (**C, D**) Comparison between (**C**) the conventional and (**D**) the new-found route for activation of oxidoreductases. ED: electron donor. EM: electron mediator.
